# Secondary prevention of stroke. A telehealth-delivered physical activity and diet pilot randomized trial (ENAbLE-pilot)

**DOI:** 10.1177/17474930231201360

**Published:** 2023-09-29

**Authors:** Coralie English, Emily R Ramage, John Attia, Julie Bernhardt, Billie Bonevski, Meredith Burke, Margaret Galloway, Graeme J Hankey, Heidi Janssen, Richard Lindley, Elizabeth Lynch, Chris Oldmeadow, Catherine M Said, Neil J Spratt, Karly Zacharia, Lesley MacDonald-Wicks, Amanda Patterson

**Affiliations:** 1 School of Health Sciences, University of Newcastle, Newcastle, NSW, Australia; 2 Heart and Stroke Program, Hunter Medical Research Institute, Newcastle, NSW, Australia; 3 Centre of Research Excellence to Accelerate Stroke Trial Innovation and Translation, University of Sydney, Sydney, NSW, Australia; 4 Allied Health, Western Health, Melbourne, VIC, Australia; 5 School of Medicine and Public Health, University of Newcastle, Newcastle, NSW, Australia; 6 Division of Medicine, John Hunter Hospital, Hunter New England Local Health District, Newcastle, NSW, Australia; 7 The Florey Institute, Melbourne, VIC, Australia; 8 Flinders Health and Medical Research Institute, College of Medicine and Public Health, Flinders University, Adelaide, SA, Australia; 9 Medical School, Faculty of Health and Medical Sciences, The University of Western Australia, Perth, WA, Australia; 10 Perron Institute for Neurological and Translational Science, Perth, WA, Australia; 11 Hunter Stroke Services, Hunter New England Local Health District, Newcastle, NSW, Australia; 12 Westmead Applied Research Centre, Sydney Medical School, University of Sydney, Sydney, NSW, Australia; 13 Caring Futures Institute, Flinders University, Adelaide, SA, Australia; 14 Hunter Medical Research Institute, Newcastle, NSW, Australia; 15 Physiotherapy, Western Health, St Albans, VIC, Australia; 16 Physiotherapy, University of Melbourne, Parkville, VIC, Australia; 17 School of Biomedical Sciences and Pharmacy, University of Newcastle, Newcastle, NSW, Australia; 18 Department of Neurology, John Hunter Hospital, Hunter New England Local Health District, Newcastle, NSW, Australia; 19 Food and Nutrition Program, Hunter Medical Research Institute, Newcastle, NSW, Australia

**Keywords:** Physical activity, diet, telehealth, pilot trial, blood pressure, quality of life

## Abstract

**Background:**

Improving physical activity levels and diet quality are important for secondary stroke prevention.

**Aim:**

To test the feasibility and safety of 6-month, co-designed telehealth-delivered interventions to increase physical activity and improve diet quality.

**Methods:**

A 2 × 2 factorial trial (physical activity (PA); diet (DIET); PA + DIET; control) randomized, open-label, blinded endpoint trial. Primary outcomes were feasibility and safety. Secondary outcomes included stroke risk factors (blood pressure, self-report PA (International Physical Activity Questionnaire (IPAQ)) and diet quality (Australian Recommended Food Score (ARFS)), and quality of life. Between-group differences were analyzed using linear-mixed models.

**Results:**

Over 23 months, 99 people were screened for participation and 40 (40%) randomized (3 months to 10 years post-stroke, mean age 59 (16) years). Six participants withdrew, and an additional five were lost to follow-up. Fifteen serious adverse events were reported, but none were deemed definitely or probably related to the intervention. Median attendance was 32 (of 36) PA sessions and 9 (of 10) DIET sessions. The proportion of missing primary outcome data (blood pressure) was 3% at 3 months, 11% at 6 months, and 14% at 12 months. Between-group 95% confidence intervals showed promising, clinically relevant differences in support of the interventions across the range of PA, diet quality, and blood pressure outcomes.

**Conclusion:**

Our telehealth PA and diet interventions were safe and feasible and may have led to significant behavior change.

**Trial Registration:**

ACTRN12620000189921.

## Introduction

Low levels of physical activity (PA) and poor-quality diet are among the top-10 independent risk factors for stroke and influence other important risk factors including hypertension, dyslipidemia, and obesity.^1^ International Clinical Guidelines recommend increasing PA and improving diet quality to reduce risk of recurrent stroke.^2^

Exercise interventions for people with stroke are effective for reducing blood pressure but only when supervised aerobic exercise is provided.^3^ There are no trials of dietary interventions for secondary stroke prevention, but the Mediterranean-style diet holds the most promise.^4^ Increasing PA and improving diet quality involves behavior change. Effective behavior change strategies include monitoring, tailored education, personalized goal setting, prompts, and social support.^5^ People living with stroke face additional barriers and therefore require personalized support. Transport and access to services in regional and remote areas are additional barriers. Telehealth-delivered services are a potential solution.

Our trial tested the feasibility and safety of two telehealth-delivered interventions for reducing stroke risk factors, co-designed in partnership with people with stroke, researchers and healthcare clinicians and included evidence-based behavior change strategies.

Our primary research question was:

1. Are telehealth-delivered PA and/or diet interventions *feasible* and *safe* for people with stroke or transient ischaemic attack (TIA)?

Secondary questions were:

2. Do the interventions reduce stroke risk factors (decreased blood pressure, increased PA, improved diet quality)?
3. Do the interventions improve fatigue, mood, and health-related quality of life?

## Method

### Design

A prospective 2 × 2 factorial (4 arm) pilot randomized, open-label, blinded endpoint trial was conducted in accordance with the CONSORT checklist for pilot studies.^6^ Ethics approvals obtained from the Hunter New England Human Research Ethics Committee (2019/ETH11533) and University of Newcastle Human Research Committee (H-2020-0022). Full details of the trial design are reported elsewhere.^7^ All aspects of the trial were conducted via telehealth with no in person contact.

### Participants

Participants were recruited from around Australia via referral from hospital-based stroke clinics, rehabilitation facilities, and word of mouth (including promotion via non-government organizations and social media). In brief, inclusion criteria were adults who experienced a stroke or transient ischaemic attack between 3 months and 10 years previously; living at home; able to walk independently indoors; sufficient cognitive ability (or a carer to support) to engage in the interventions. Exclusion criteria included: currently meeting PA guidelines or adhering to a Mediterranean-style diet (assessed via International Physical Activity Questionnaire (IPAQ)^8^ and Mediterranean Diet Score (MDS)^9^ respectively). Participants provided written informed consent.

### Baseline assessment

Baseline assessment included demographic information, details about participants’ stroke event, stroke severity (National Institutes of Health Stroke Scale, measured at baseline assessment), disability level (modified Rankin Scale score, Functional Ambulation Classification) and exercise self-efficacy (Short Self-efficacy for Exercise Scale). Participants were randomized with block allocation (block size of 4 prepared by an independent statistician and coded into the trial database) after baseline assessment. Further assessments occurred at 3-, 6-, and 12 months by a blinded assessor.

### Intervention

The two co-designed intervention packages were: *“i-REBOUND—Let’s get moving*” (PA intervention ); and *“i—REBOUND—Eat for health*” (DIET intervention). We describe these 6 month, telehealth-delivered interventions according to the Template for Intervention Description and Replication checklist (TIDieR) elsewhere.^10,11^ The intervention sessions were hosted via real-time video conferencing software (“Attend Anywhere” (Attend Anywhere Pty Ltd) or “Zoom” (Zoom Video Communications, Inc.)).

Both the PA and DIET interventions included evidence-based, tailored behavior change support strategies.^5^ Use of an activity monitor (provided FitBit™ or own device) or activity diaries were encouraged in the PA intervention. The DIET intervention included completion of the MDS at each DIET session to help identify new goals and strategies for adherence. Manuals were developed to train intervention therapists. Resources including information booklets, blood pressure monitors, activity monitors, recipe books and other diet support resources including a starter pack of key ingredients were mailed to participants. The four arms of the trial are described briefly below:

CONTROL: Information and general advice about PA and diet via two telehealth sessions (Months 0 and 3). Dissemination of hard copy documents (and/or links to online resources) about Australian Government PA and healthy eating guidelines.

PA: Months 0–3: Twice weekly in-home telehealth-supervised exercise sessions (24 in total). Each session included (1) supervised, real-time exercise aiming for 20 mins of moderate to vigorous PA, (target heart rate 55% to 90% of age-predicted maximum (HRMax); and/or rating of perceived exertion (Borg category ratio 10, CR-10) between 3 and 6 points), and (2) discussion of goals and strategies to support PA to achieve a minimum of 150 minutes of moderate to vigorous PA per week. Months 4 to 6: Individually tailored home and community exercise program with once-weekly telehealth support.

DIET: Months 0–3: Telehealth-delivered, individualized dietary counseling sessions in weeks 1 and 2 and fortnightly thereafter (7 in total) to support participants to follow a Mediterranean-style diet. Months 4 to 6: Monthly dietary counseling sessions.

PA + DIET: The PA and diet intervention packages were delivered simultaneously.

### Outcome measures

Our co-primary aims were feasibility and safety at 6 months. Feasibility was assessed by trial metrics including proportion of eligible people enrolled, adherence to intervention sessions, participant drop-out rates and missing data for the exploratory clinical outcomes. To assess safety, adverse event data were collected via telehealth at each assessment time point, and during intervention sessions (7). An independent Data Safety Monitor adjudicated adverse event data. Blood pressure was measured using the iHealth Track (KN-550BT) monitor, mailed to participants and collected in accordance with recommended guidelines. Other secondary measures collected via telehealth included self-reported PA (IPAQ), diet quality (Australian Recommended Food Score (ARFS) derived from the Australian Eating Survey,^12^ fatigue (Fatigue Severity Scale, 7-item), mood (Depression, Anxiety and Stress Scale, 21-item) and quality of life (EuroQol-5 Dimensions). Our process evaluation (see)^7^ will be reported separately.

### Data analyses

Participant demographics, feasibility, and safety data were analyzed descriptively. To explore the interventions’ effect on stroke risk factors, fatigue mood, and quality of life, we evaluated within- and between-group effects using linear-mixed models, which included baseline, mid-intervention (3 month), end-intervention (6 month), and long-term follow-up (12 month) measures with *p* values < 0.05 considered statistically significant. Mixed-model analysis included imputation of missing data and adjustments for multiple comparisons. We assumed no interaction between the interventions^13^ and collapsed groups (supplemental Figure 1) to examine the individual effects of PA and diet on stroke risk factors, fatigue, mood, and quality of life as follows:

1. PA and PA + DIET compared with CONTROL and DIET (exploring effect of PA intervention).
2. DIET and PA + DIET compared with CONTROL and PA (exploring effect of DIET intervention).

As this was a pilot trial, formal sample size calculations were not conducted. We aimed to recruit 80 participants, with an interim target of *n* = 40.

## Results

**Figure 1.**
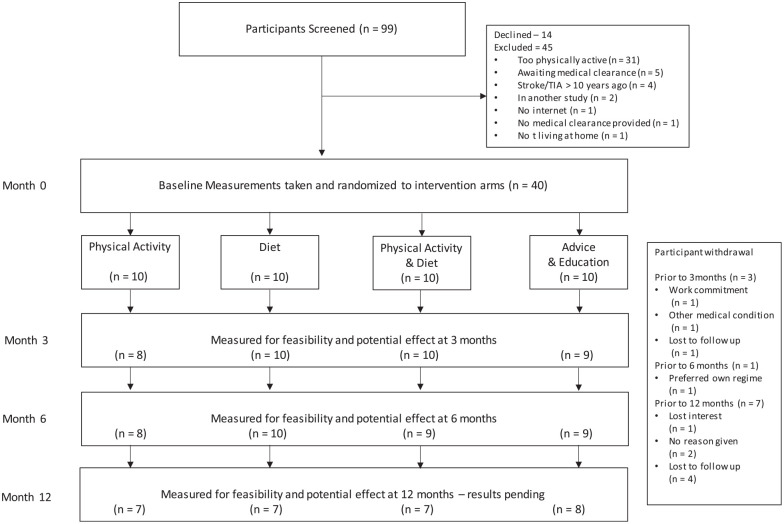
Design and flow of participants through the trial.

Figure 1 presents the flow of participants through the trial. Table 1 presents baseline characteristics. Mean participant age was 59 (SD 16) years, 16 (40%) were female, and the median time since stroke was 39 (interquartile range (IQR) 7–63) months. There were no substantial differences in characteristics between participants retained and lost to follow-up at 12 months.

**Table 1.** Baseline characteristics of participants.

Characteristic	Randomized*	Lost to follow-up
	(n = 40)	(n = 11)
Age (year), mean (SD)	59 (16)	58 (17)
Sex, n females (%)	16 (40)	4 (37)
Support person available, n (%)	32 (80)	9 (82)
Interpreter required, n (%)	0 (0)	0 (0)
Born in Australia, n (%)	33 (83)	8 (73)
Aboriginal or Torres Strait Islander***	1 (3)	0 (0)
Level of education		
Did not complete high school	10 (25)	4 (36)
High school/trade/certificate/diploma	21 (53)	4 (36)
University degree	9 (22)	3 (27)
Aphasia**		
No aphasia	37 (92)	10 (91)
Mild to moderate aphasia	3 (8)	1 (9)
Rural, n (%)	11 (28)	3 (27)
Comorbidities, n (%)		
Hypertension (self or GP reported)	26 (65)	8 (73)
High cholesterol	22 (55)	6 (55)
Diabetes	4 (10)	1 (9)
Atrial fibrillation	3 (8)	2 (18)
Ischaemic heart disease	2 (5)	0 (0)
Other cardiac conditions	8 (20)	4 (36)
Other	31 (78)	8 (73)
Nil comorbidities	1 (3)	0 (0)
Taking beta blocker medication, n (%)	0 (0)	0 (0)
Smoker, n (%)	4 (10)	1 (10)
Time (months) since first stroke/TIA, median (IQR)	39 (7 to 63)	15 (7 TO 63)
Side most affected by stroke, n (%)		
Left	21 (53)	6 (55)
Right	11 (27)	3 (27)
Neither	6 (15)	2 (18)
Both	2 (5)	0 (0)
Walks without aid, n (%)	19 (95)	11 (100)
>1 self-reported fall in the last year, n (%)	29 (73)	3 (27)
NIHSS, n (%)		
Mild (0–7)	40 (100)	11 (100)
MRS, n (%)		
No symptoms	6 (15)	1 (9)
No significant disability	22 (55)	9 (82)
Slight disability	6 (15)	0 (0)1 (9)
Moderate disability	6 (15)	
FAC, n (%)		
5 = Independent ambulator	35 (87)	10 (91)
4 = Independent ambulator level surfaces	3 (7)	1 (9)
3 = Dependent for supervision	1 (3)	0 (0)
1 or 2 = Dependent for physical assistance	1 (3)	0 (0)
SEE (0–90) mean (SD)	68 (15)	74 (11)

TIA: transient ischemic attack; IQR: interquartile range; NIHSS: National Institute of Health Stroke Scale; MRS: modified Rankin Scale; FAC: Functional Ambulation Classification; SEE: Self-Efficacy for Exercise scale.

* Lost to follow-up at any time point.

One intervention therapist (physiotherapist, 15 years’ experience) provided the majority (334 (63%) of PA intervention sessions. The remainder were provided by another physiotherapist (20 years’ experience), and an exercise scientist (28 years’ experience). All DIET intervention sessions were provided by the same dietitian (5 years’ experience).

### Feasibility

Between March 2020 and January 2022, we screened 99 people: 45 were ineligible, 14 declined, and 40 were randomized (Figure 1). The most common reason for ineligibility was being too physically active (n = 31). Six participants withdrew from the trial, and an additional 5 participants were lost to follow-up (Figure 1). The proportion of missing data for the clinical outcome of blood pressure was 3% at 3 months, 11% at 6 months, and 14% at 12 months (Table 2).

**Table 2.** Feasibility measures.

Percentage of eligible participants recruited, n (%)	40 (41)			
Time from screening to randomization, median (IQR), (days)	29 (22 to 34)			
Home visit required, n (%)	0 (0)			
Number of PA sessions attended*, median (IQR), target, n = 36	32 (26 to 34)			
Number of DIET sessions attended, median (IQR), target, n = 10	9 (8 to 10)			
Number of sessions not completed due to technology failure, n (%)	0 (0)			
% of age-predicted HR during supervised PA sessions (beats/min), mean (SD)	65 (7)			
Borg rating of perceived exertion during supervised PA sessions (score), median (IQR)	3.3 (2.9 to 4.3)			
Duration of supervised exercise sessions (min), mean (SD)	49 (9)			
Time spent exercising during supervised sessions (min), mean (SD)	20 (0.4)			
Duration of completed DIET sessions (min), mean (SD)	53 (15)			
Number of participants with complete outcome data, n (%)	Baseline (n = 40)	3 months (n = 37)	6 months (n = 36)	12 months (n = 29)
Valid BP data (>3 days or 6 measures)	39 (98)	36 (97)	32 (89)	25 (86)
ActivPAL devices collected	38 (95)	34 (92)	34 (94)	27 (93)
Questionnaires completed**	40 (100)	35 (95)	36 (100)	29 (100)

* (NB—supervised + support sessions); **Included International Physical Activity Questionnaire, Mediterranean Diet Score, Depression and Anxiety Stress Scale 21, European Quality of Life Scale 5-D, Fatigue Severity Scale (7-item).

A total of 527 (73% of scheduled) PA sessions were attended. During supervised exercise sessions, participants, on average, exercised for 20 (SD 0.4) minutes, reached 65% (SD 7) of their predicted HRmax, and had a perceived exertion rating of 3.4 (SD 1.0) on the Borg CR-10 scale (Table 2). A total of 182 (91% of scheduled) diet sessions were attended.

### Safety

Fifteen serious adverse events occurred (mostly hospitalization for unrelated health issues); none were deemed definitely or probably related to the trial. There were 85 adverse events; 25 were deemed possibly or probably related, and 5 (light-headedness, pain) were deemed definitely related to the intervention. No falls occurred during supervised exercise sessions. See Supplemental Tables.

### Effect of the PA intervention on stroke risk factors, fatigue, mood, and quality of life

Table 3 presents results for the effect of the PA intervention on stroke risk factors, fatigue, mood, and quality of life. There were no significant differences for changes in blood pressure over time. There was, however, a trend toward greater systolic and diastolic blood pressure reductions at 3 months in the PA group compared with CONTROL (mean between-group difference (95% CI) −4.1 mmHg (−9.1 to 1.0) and −2.7 mmHg (−5.4 to 0.1), respectively).

**Table 3.** Effect of physical activity on stroke risk factors, fatigue, mood, and health-related quality of life (values are mean (SD), or estimated mean difference (95% confidence interval; bold indicates statistical significance).

Outcome	Time point								Difference within groups						Difference between groups		
Groups	Month 0		Month 3		Month 6		Month 12		Month 3 − Month 0		Month 6 − Month 0		Month 12 − month 0		Month 3	Month 6	Month 12
	Exp	Con	Exp	Con	Exp	Con	Exp	Con	Exp	Con	Exp	Con	Exp	Con	Exp–Con	Exp–Con	Exp–Con
Blood pressure, systolic (mmHg)	128 (11)	131 (12)	127 (7)	133 (13)	125 (10)	128 (11)	127 (11)	129 (16)	–1.1 (–5.7 to 3.4)	2.9 (–1.5 to 7.3)	–2.3 (–6.8 to 2.3)	–1.4 (–5.8 to 3.1)	–1.4 (–6.3 to 3.4)	–1.6 (–6.2 to 3.1)	–4.1 (–9.1 to 1.0)	–0.9 (–6.1 to –4.4)	0.1 (–5.5 to 5.8)
Blood pressure, diastolic (mmHg)	80 (7)	84 (7)	78 (6)	84 (9)	77 (7)	83 (7)	78 (6)	81 (7)	–2.5 (–4.9 to 0.0 0.01)	0.2 (–2.2 to 2.6)	–1.9 (–4.4 to –0.6)	–1.0 (–3.4 to –1.4)	–1.4 (–4.0 to 1.3)	–2.4 (–4.9 to 0.2)	–2.7 (–5.4 to 0.1)	–0.9 (–3.8 to 1.9)	1.0 (–2.1 to 4.0)
IPAQ (METmin.wk)	434 (428)	497 (597)	1650 (1225)	967 (695)	1528 (1359)	1032 (1477)	1712 (1457)	748 (667)	**1227 (551 to 1903)**	**483 (0 to 965)**	**1277 (404 to 2149)**	726 (–288 to 1741)	**1511 (625 to 2397)**	440 (–61 to 942)	**744 (73 to 1415)**	550 (–32 to 1421)	**1071 (271 to 1871)**
FSS-7 (score)	37 (13)	42 (14)	34 (14)	37 (13)	32 (12)	34 (14)	38 (9)	41 (13)	–2.1 (–9.6 to 5.4)	–4.3 (–11.6 to 3.0)	–4.8 (–12.5 to 3.0)	–6.1 (–13.4 to 1.2)	2.3 (–5.7 to 10.1)	2.2 (–5.4 to 9.9)	2.1 (–6.2 to 10.5)	1.4 (–7.2 to 9.8)	0.6 (–7.2 to 8.3)
DASS—depression (score)	3.9 (4.9)	3.4 (3.7)	3.8 (5.8)	3.8 (5.8)	2.9 (3.8)	2.9 (3.8)	4.1 (5.5)	4.1 (5.6)	–0.4 (–2.4 to 1.7)	0.9 (–1.2 to 2.9)	–0.9 (–2.9 to 1.2)	0.4 (–1.7 to 2.4)	–0.2 (–2.2 to 1.9)	1.8 (–0.3 to 3.8)	–1.2 (–3.5 to 1.1)	–1.2 (–3.5 to 1.1)	–1.9 (–4.2 to 0.4)
DASS—anxiety (score)	3.0 (3.5)	1.9 (2.1)	3.0 (5.1)	2.4 (3.0)	1.6 (2.9)	1.1 (1.7)	2.0 (3.2)	0.8 (1.4)	0.2 (–1.3 to 1.7)	0.8 (–0.7 to 2.3)	–0.9 (–2.4 to 0.6)	–0.3 (–1.8 to 1.2)	–0.7 (–2.2 to 0.8)	–0.7 (–2.2 to 0.8)	–0.6 (–2.3 to 1.2)	–0.7 (–2.4 to 1.1)	0.5 (–1.7 to 1.8)
DASS—stress (score)	5.6 (4.0)	4.2 (4.2)	5.1 (5.2)	3.8 (4.0)	3.5 (3.6)	2.6 (3.4)	3.9 (3.8)	3.6 (4.6)	0.2 (–1.5 to 1.9)	0.3 (–1.5 to 2.0)	–1.0 (–2.8 to 0.8)	–0.5 (–2.3 to 1.2)	–0.5 (–2.2 to 1.3)	0.7 (–1.1 to 2.4)	–0.1 (2.1 to 1.9)	–0.5 (–2.5 to 1.5)	–1.1 (–3.1 to 0.9)
EQ-5D mobility (score, range 1–5))	1.3 (0.6)	1.6 (0.9)	1.4 (0.7)	1.7 (0.9)	1.2 (0.6)	1.8 (1.0)	1.2 (0.5)	1.7 (0.9)	0.1 (–0.2 to 0.4)	0.1 (–0.2 to 0.4)	–0.2 (–0.5 to 0.1)	0.2 (–0.1 to 0.5)	–0.1 (–0.4 to 0.2)	0.03 (–0.3 to 0.3)	2.0 (–0.3 to 0.3)	**0.4 (0.01 to 0.7)**	0.2 (–0.2 to 0.5)
EQ-5D—self care (score, range 1–5)	1.3 (0.6)	1.5 (1.0)	1.2 (0.4)	1.5 (0.7)	1.2 (0.5)	1.5 (0.7)	1.2 (0.5)	1.5 (0.8)	–0.02 (–0.3 to 0.2)	0.1 (–0.1 to 0.3)	–0.1 (–0.3 to 0.2)	0.1 (–0.1 to 0.4)	–0.04 (–0.3 to 0.2)	0.1 (–0.2 to 0.4)	0.1 (–0.2 to 0.4)	0.2 (–0.1 to 0.5)	0.1 (–0.2 to 0.5)
EQ-5D—usual activities (score, range 1–5))	1.4 (0.6)	1.9 (1.1)	1.5 (0.9)	1.8 (1.1)	1.5 (0.7)	2.0 (1.0)	1.4 (0.6)	1.9 (0.9)	0.1 (–0.4 to 0.6)	–0.2 (–0.7 to 0.3)	0.2 (–0.4 to 0.7)	0.2 (–0.3 to 0.7)	–0.04 (–0.6 to 0.5)	–0.1 (–0.7 to 0.4)	–0.3 (–0.9 to 0.3)	0.03 (–0.6 to 0.6)	–0.1 (–0.4 to 0.9)
EQ-5D—Pain (score, range 1–5))	1.5 (0.8)	1.8 (0.9)	1.5 (0.6)	1.8 (0.9)	1.4 (0.7)	2.1 (0.9)	1.5 (0.8)	2.1 (0.9)	–0.1 (–0.5 to 0.3)	–0.1 (–0.5 to 0.3)	–0.3 (–0.7 to 0.1)	–0.1 (–0.3 to 0.5)	–0.3 (–0.7 to 0.1)	–0.1 (–0.5 to 0.3)	–0.1 (–0.5 to 0.4)	0.4 (–0.1 to 0.8)	0.2 (–0.3 to 0.7)
EQ-5D—Anxiety/depression (score, range 1–5))	1.7 (0.9)	1.4 (0.7)	1.5 (0.6)	1.8 (0.9)	1.4 (0.7)	2.1 (0.9)	1.5 (0.8)	2.1 (0.9)	–0.02 (–0.4 to 0.4)	0.1 (–0.4 to 0.5)	0.1 (–0.4 to 0.5)	0.2 (–0.2 to 0.6_	0.1 (–0.4 to 0.6)	**0.5 (<** **0.01 to 0.9)**	0.1 (–0.4 to 0.6)	0.1 (–0.4 to 0.6)	0.4 (–0.2 to 0.9)
EQ-5D—health today (score, range 0 to 100)	78 (14)	68 (20)	81 (13)	71 (16)	82 (9)	69 (14)	80 (12)	69 (17)	–0.04 (–8.0 to 7.9)	–1.3 (–9.0 to 6.5)	–2.6 (–10.5 to 5.4)	–4.5 (–12.2 to 3.2)	7.0 (–1.6 to 15.6)	3.3 (–5.3 to 11.9)	–0.7 (–9.0 to 7.9)	–1.5 (–9.8 to 6.9)	–3.0 (–11.1 to 5.3)

Exp: physical activity (PA and PA + DIET groups combined); Con: no physical activity (CONTROL and DIET groups combined); IPAQ: International Physical Activity Questionnaire; FSS-7: Fatigue Severity Scale–7 item; DASS: Depression, Anxiety and Stress Scale; EQ-5D: European Quality of Life Scale.

Participants who received the PA intervention reported spending significantly more time physically active at 3 months, compared with participants who did not (mean between-group difference 744 METmin/wk (95% CI: 73–1415)). This between-group difference was lost at 6 months but was present again at 12 months (mean between-group difference 1071 METmin/wk (95% CI: 271–1871)). Data for device-measured PA will be reported in future papers.

The PA intervention did not have a significant effect on self-reported fatigue, mood, or quality of life.

### Effect of the diet intervention on stroke risk factors, fatigue, mood, and quality of life

Table 4 presents results for the effect of the DIET intervention on stroke risk factors, fatigue, mood, and quality of life. Participants receiving the DIET intervention had significantly lower diastolic blood pressure at 6 months compared with baseline (mean within group difference DIET −2.6 mmHg (95% CI: −5.0 to −0.2). There were no other significant within or between-group differences for changes in blood pressure over time.

**Table 4.** Effect of diet on stroke risk factors, fatigue, mood and health-related quality of life (values are mean (SD), or estimated mean difference (95% confidence interval; bold indicates statistical significance).

Outcome	Time point								Difference within groups						Difference between groups		
	Month 0		Month 3		Month 6		Month 12		Month 3 minus Month 0		Month 6 minus Month 0		Month 12 minus month 0		Month 3	Month 6	Month 12
Groups	Exp	Con	Exp	Con	Exp	Con	Exp	Con	Exp	Con	Exp	Con	Exp	Con	Exp − Con	Exp − Con	Exp − Con
Blood pressure, systolic (mmHg)	132 (12)	128 (11)	131 (11)	129 (11)	128 (10)	126 (11)	130 (16)	126 (10)	1.2 (–3.1 to 5.4)	2.9 (–1.5 to 7.3)	–3.6 (–8.0 to 0.8)	–1.4 (–5.8 to 3.1)	–1.6 (–6.6 to 3.4)	–1.6 (–6.2 to 3.1)	–1.8 (–6.8 to 3.3)	–2.3 (–7.5 to 3.0)	0.0 (–5.7 to 5.6)
Blood pressure, diastolic (mmHg)	82 (7)	82 (8)	82 (9)	80 (7)	80 (7)	80 (7)	80 (6)	80 (7)	0.8 (–1.6 to 3.1)	0.2 (–2.2 to 2.6)	**–2.6 (–5.0 to –0.2)**	–1.0 (–3.4 to 1.4)	–2.7 (–5.4 to 0.0)	–2.4 (–4.9. to 0.2)	0.6 (–2.2 to 3.3)	–1.6 (–4.4 to 1.3)	–0.3 (–3.4 to 2.8)
MDS (score)(1 to 14)	4.7 (1.4)	5.3 (1.4)	7.9 (2.5)	5.4 (1.7)	8.2 (2.5)	5.6 (1.1)	6.5 (2.7)	5.7 (1.7)	**3.1 (2.1 to 4.1)**	–6.2 (–1.0 to 1.0)	**3.5 (2.4 to 4.5)**	0.3 (–0.8 to 1.3)	**1.8 (0.7 to 2.8)**	0.4 (–0.7 to 1.4	**3.1 (1.9 to 4.3)**	**3.2 (2.0 to 4.4)**	**1.4 (0.2 to 2.6)**
ARFS (score)(0 to 76)	29.5 (9.7)	32.0 (9.7)	35.8 (7.8)	32.0 (8.7)	35.5 (7.0)	33.9 (7.5)	33.4 (9.0)	31.7 (10.4)	**6.6 (3.1 to 10.0)**	–0.2 (–3.8 to 3.4)	**7.9 (4.4 to 11.3)**	3.3 (–0.3 to 6.8)	**3.9 (0.0 to 7.7)**	–0.3 (–4.1 to 3.4)	**6.8 (2.7 to 10.9)**	**4.6 (0.5 to 8.8)**	4.2 (–0.3 to 8.6)
FSS–7 (score)	42 (13)	37 (14)	37 (13)	34 (16)	33 (12)	33 (15)	40 (11)	38 (11)	–6.3 (–13.4 to 0.8)	–4.3 (–11.6 to 3.0)	**–9.5 (–16.6 to –2.3)**	–6.1 (–13.4 to 1.2)	1.3 (–6.6 to 9.2)	2.2 (–5.4 to 9.9)	2.0 (–10.5 to 6.4)	–3.3 (–11.9 to 5.2)	–0.8 (–10.0 to 8.1)
DASS—depression (score)	4.5 (3.9)	2.8 (4.5)	3.9 (6.0)	3.1 (5.4)	2.5 (3.7)	2.6 (4.6)	3.2 (3.8)	3.6 (6.3)	–0.1 (–2.1 to 2.0)	0.9 (–1.2 to 2.9)	–1.5 (–3.5 to 0.6)	0.4 (–1.7 to 2.4)	–0.4 (–2.4 to 1.7)	1.8 (–0.3 to 3.8)	–0.9 (–3.2 to 1.4)	–1.8 (–4.1 to 0.54)	–2.1 (–4.4 to 0.24)
DASS—anxiety (score)	3.0 (3.5)	1.9 (2.1)	3.0 (5.1)	2.4 (3.0)	1.6 (3.0)	1.1 (1.7)	2.0 (3.2)	0.8 (1.4)	0.2 (–1.3 to 1.7)	0.8 (–0.7 to 2.3)	–1.2 (–2.7 to 0.3)	–0.3 (–1.8 to 1.2)	–1.4 (–2.9 to 0.1)	–0.7 (–2.2 to 0.8)	–0.6 (–2.3 to 1.2)	–1.0 (2.7 to 0.8)	–0.7 (–2.4 to 1.1)
DASS—stress (score)	6.0 (4.2)	3.9 (3.8)	4.7 (4.7)	4.1 (4.7)	3.0 (3.4)	3.1 (3.6)	3.6 (3.6)	4.0 (4.8)	–1.2 (–3.0 to 0.6)	0.3 (–1.4 to 2.0)	**–2.7 (–4.5 to –1.0)**	–0.5 (–2.2 to 1.2)	**–1.9 (–3.6 to –0.1)**	0.7 (–1.1 to 2.4)	–1.5 (–3.5 to 0.5)	–2.2 (–4.2 to –0.2)	–2.5 (–4.5 to –0.5)
EQ5–D—mobility (score, range 1–5)	1.5 (0.7)	1.4 (0.8)	1.6 (0.7)	1.5 (0.9)	1.5 (0.8)	1.6 (0.9)	1.4 (0.8)	1.4 (0.7)	0.1 (–0.2 to 0.4)	0.1 (–0.2 to 0.4)	–0.2 (–0.5 to 0.1)	–0.03 (–0.3 to 0.3)	–0.1 (–0.4 to 0.2)	–0.1 (–0.4 to 0.2)	1.2 (–0.3 to 0.3)	0.2 (–0.2 to 0.5)	0.1 (–0.3 to 0.4)
EQ5–D—self care (score, range 1–5)	1.3 (0.6)	1.5 (1.0)	1.3 (0.5)	1.4 (0.7)	1.3 (0.7)	1.4 (0.8)	1.3 (0.4)	1.4 (0.8)	–0.02 (–0.3 to 0.2)	–0.2 (–0.5 to 0.1)	–0.1 (–0.3 to 0.2)	0.2 (–0.5 to 0.03)	–0.04 (–0.3 to 0.2)	0.2 (–0.5 to 0.1)	–0.2 (–0.5 to 0.1)	–0.1 (–0.4 to 0.1)	–0.2 (–0.5 to 0.1)
EQ5–D—usual activities (score, range 1–5)	1.7 (0.9)	1.5 (0.9)	1.7 (0.8)	1.6 (1.1)	1.9 (0.9)	1.6 (0.9)	1.6 (0.8)	1.47(0.8)	0.1 (–0.4 to 0.6)	0.3 (–0.2 to 0.8)	0.2 (–0.4 to 0.7)	0.1 (–0.4 to 0.7)	–0.04 (–0.6 to 0.5)	0.2 (–0.3 to 0.8)	0.2 (–0.4 to 0.7)	–0.1 (–0.6 to 0.5)	–0.3 (–0.3 to 0.8)
EQ5–D—Pain (score, range 1–5)	1.8 (0.9)	1.5 (0.8)	1.7 (0.9)	1.6 (0.7)	1.7 (0.9)	1.8 (0.8)	1.6 (0.8)	1.9 (1.0)	–0.1 (–0.5 to 0.3)	0.2 (–0.2 to 0.6)	–0.3 (–0.7 to 0.1)	0.2 (–0.2 to 0.6)	–0.3 (–0.7 to 0.1)	**0.4 (0.01 to 0.8)**	0.3 (–0.2 to 0.7)	**0.5 (0.0 to 0.90**	**0.7 (0.2 to 1.1)**
EQ5–D—Anxiety/depression (score, range 1–5)	1.5 (0.8)	1.6 (0.8)	1.6 (0.8)	1.7 (0.9)	1.7 (0.8)	1.5 (0.8)	1.7 (0.9)	1.6 (0.9)	–0.02 (–0.4 to 0.4)	0.02 (–0.4 to 0.5)	0.1 (–0.4 to 0.5)	–0.2 (–0.6 to 0.3)	0.1 (–0.4 to 0.6)	–0.2 (–0.7 to 0.2)	0.04 (–0.5 to 0.5)	–0.3 (–0.7 to 0.2)	–0.3 (–0.9 to 0.2)
EQ5–D—Health today (score, range 0–100)	74 (10)	72 (24)	73 (15)	78 (15)	70 (12)	79 (13)	77 (11)	75 (18)	–0.04 (–8.0 to 7.9)	–1.3 (–9.0 to 6.5)	–2.6 (–10.5 to 5.4)	–4.5 (–12.2 to 3.2)	7.0 (–1.6 to 15.6)	3.3 (–5.3 to 11.9)	**6.8 (1.5 to 15.1)**	**10.1 (1.8 to 18.4)**	–0.6 (–8.9 to 7.7)

Exp: diet (DIET and PA + DIET groups combined); Con: no diet (CONTROL and PA groups combined); MDS: Mediterranean Diet Score; ARFS: Australian Recommended Food Score; FSS-7: Fatigue Severity Scale–7 item; DASS: Depression, Anxiety and Stress Scale; EQ-5D: European Quality of Life.

Participants who received the DIET intervention showed promising improvement in overall diet quality at 3, 6, and 12 months, compared with baseline (mean within group differences ARFS scores 6.6 (95% CIL 3.1–10), 7.9 (4.4–11.3) and 3.9 (0–7.7), respectively). Compared with those who did not, participants who received the DIET intervention showed improvement in overall diet quality at 3 months (mean between-group difference ARFS score 6.8 points (95% CI: 6.7–10.9)) and at 6 months (mean between-group difference ARFS score 4.6 points (95% CI: 0.5–8.8)), but this effect was lost at 12 months. Detailed nutrient intake analyses will be reported in future papers.

Participants who received the DIET intervention reported improvement in quality of life at 3 and 6 months (mean between-group difference EQ5-D score 6.8 points (95% CI: 1.5–15.1)), and 10.1 points (CI: 1.8–18.4), respectively), but this effect was lost at 12 months.

Data on tolerability of the interventions and participant and therapist feedback will be reported as part of the process evaluation in future papers.

## Discussion

Telehealth-delivered PA and diet interventions were feasible and safe to deliver, and led to statistically significant, and clinically meaningful improvements in PA, diet quality, and quality of life. While not powered to detect statistically significant between-group differences, we also found a trend toward lower blood pressure associated with the diet intervention.

Conducting a complex clinical trial for people with stroke entirely via telehealth is novel, and we found it to be feasible. Telehealth delivery was not a barrier to recruitment; only one person was unable to participate due to inadequate Internet connection. Adherence to the intervention sessions was high, and percentage of missing outcome data low. Recruitment was our greatest challenge; we were unable to reach our target sample of n = 80 within the funding and time available. While not quantifiable, the pandemic likely impacted recruitment rates via hospital-based stroke clinics and rehabilitation services. Like many other trials of health behavior change, the people included in our trial lacked diversity and may not represent those at greatest need for the interventions. Most were young, lived in metropolitan areas, were normotensive and had a moderate to high degree of self-efficacy for exercise at baseline. We also had to exclude a large number of people who were already sufficiently physically active. Future trials should include strategies for recruitment to ensure people in most need are represented.

Our safety and feasibility trial was not powered to detect statistically significant results. However, both the PA and DIET interventions led to improvements in PA levels and diet quality, respectively. This, combined with positive trends in blood pressure, suggest our interventions may reduce cardiovascular disease and recurrent stroke risk. Therefore, we plan to further test these interventions within a fully powered trial.

### Strengths and limitations

Our trial was robustly designed, adhered to the CONSORT statement for pilot studies and followed international recommendations for developing and reporting complex interventions.^14^ The drop-out rate of 10% at 6 months was in line with other complex clinical behavioral intervention trials. The 28% drop-out rate at 12 months may be attributable to the lack of participant contact after 6 months.

A strength of our trial was the comprehensive reporting and adjudication of adverse events. No serious and only five non-serious adverse events were “definitely related” to the interventions. Most adverse events were reported in the PA and PA + DIET groups, where contact between trial staff and participants was more frequent. In several instances of unrelated adverse events, participants were advised to seek medical review, which led to subsequent intervention. Many people with stroke in Australia do not have routine follow-up with stroke specialists after 3 months, so it is possible that by providing a regular point of contact with health professionals, our trial may have led to benefits for participants beyond the intent of the interventions themselves.

## Conclusion

Providing telehealth exercise and diet interventions without in-person contact is safe, feasible, and may lead to reductions in important stroke risk factors. Further work to determine whether these interventions can be scaled, and delivered to a more diverse group of people with stroke is needed.

## Supplemental Material

sj-docx-1-wso-10.1177_17474930231201360 – Supplemental material for Secondary prevention of stroke. A telehealth-delivered physical activity and diet pilot randomized trial (ENAbLE-pilot) Click here for additional data file. Supplemental material, sj-docx-1-wso-10.1177_17474930231201360 for Secondary prevention of stroke. A telehealth-delivered physical activity and diet pilot randomized trial (ENAbLE-pilot) by Coralie English, Emily R Ramage, John Attia, Julie Bernhardt, Billie Bonevski, Meredith Burke, Margaret Galloway, Graeme J Hankey, Heidi Janssen, Richard Lindley, Elizabeth Lynch, Chris Oldmeadow, Catherine M Said, Neil J Spratt, Karly Zacharia, Lesley MacDonald-Wicks and Amanda Patterson in International Journal of Stroke
