# Supplementary material for: Secondary prevention of stroke. A telehealth-delivered physical activity and diet pilot randomized trial (ENAbLE-pilot)
Source: Int J Stroke. 2023 Sep 29;19(2):199–208. doi: 10.1177/17474930231201360 (PMC10811968; doi:10.1177/17474930231201360)
Supplement: sj-docx-1-wso-10.1177_17474930231201360 – Supplemental material for Secondary prevention of stroke. A telehealth-delivered physical activity and diet pilot randomized trial (ENAbLE-pilot) [file sj-docx-1-wso-10.1177_17474930231201360.docx]

Supplemental Figure 1. Parallel design with 2 groups collapsed.


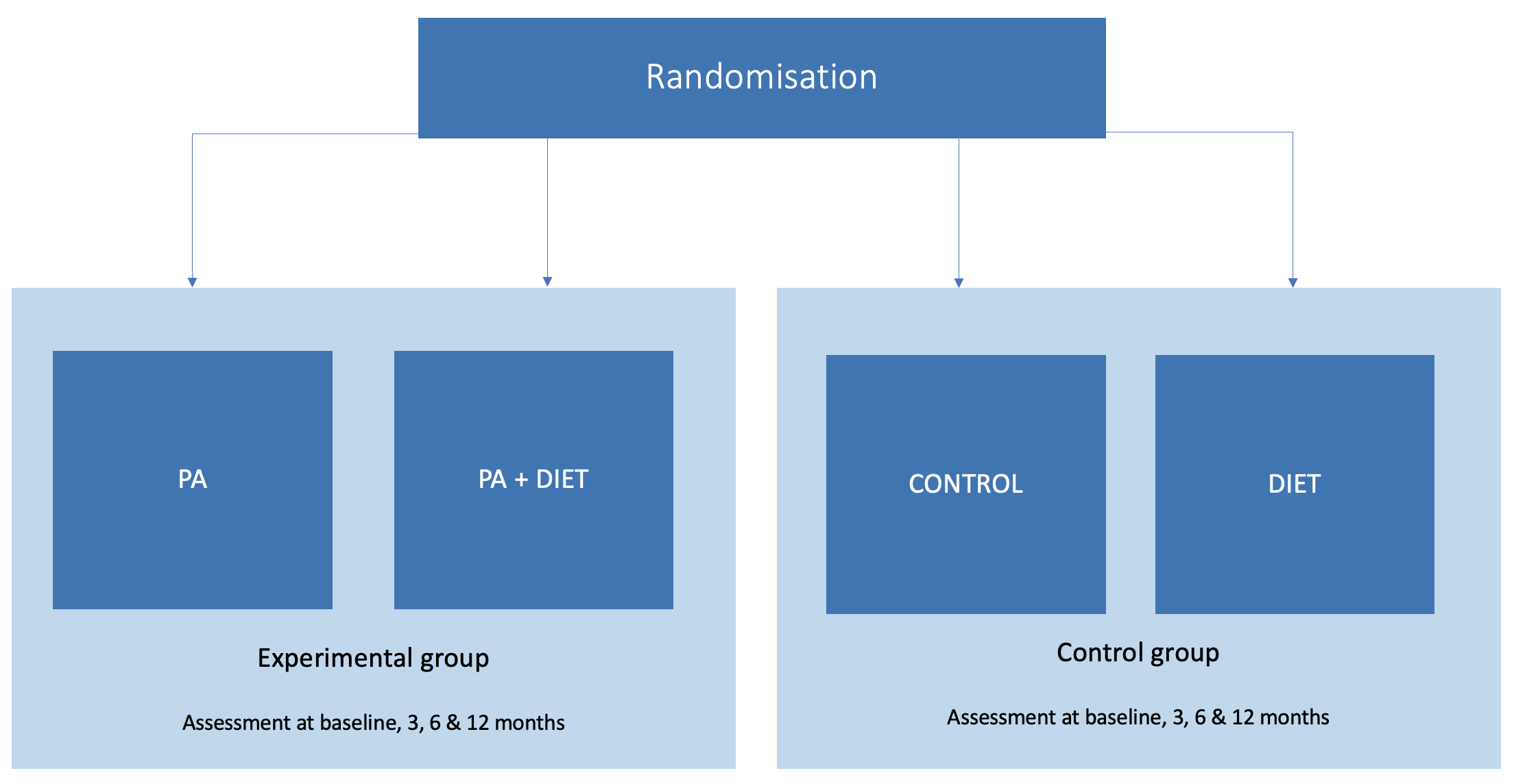


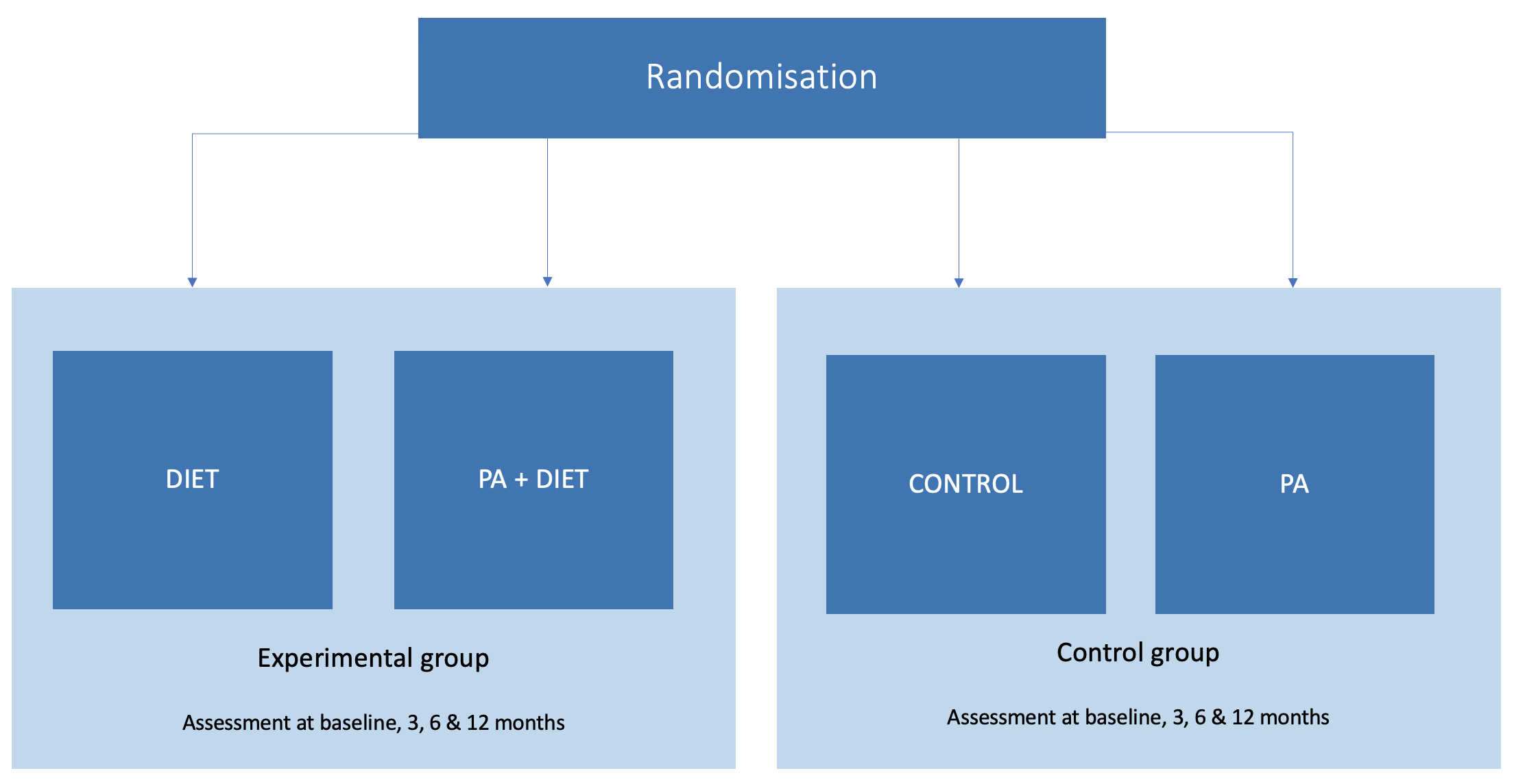


Supplemental Table 1 Adverse events

|  | Serious adverse events | | | | Adverse events | | | |
| --- | --- | --- | --- | --- | --- | --- | --- | --- |
| Causative relationship to the intervention | Control | PA | DIET | PA+DIET | Control | PA | DIET | PA+DIET |
| Definitely related | 0 | 0 | 0 | 0 | 0 | 3 | 0 | 2 |
| Probably related | 0 | 0 | 0 | 0 | 0 | 7 | 0 | 4 |
| Possibly related | 0 | 0 | 0 | 2 | 0 | 6 | 0 | 3 |
| Unlikely related | 0 | 2 | 1 | 0 | 1 | 7 | 0 | 7 |
| Not related | 2 | 4 | 1 | 3 | 3 | 13 | 5 | 20 |
| Unsure | 0 | 0 | 0 | 0 | 0 | 0 | 1 | 3 |
| **TOTAL** | **2** | **6** | **2** | **5** | **4** | **36** | **6** | **39** |

PA = physical activity intervention; DIET = diet intervention

Supplemental Table 2

Recorded Serious Adverse Events

| **Category of SAE** | **Nature of SAE** | **Relatedness to the intervention** |
| --- | --- | --- |
| Other medically important condition | Unconfirmed TIA | Possible |
| Other medically important condition | Unconfirmed TIA | Possible |
| Hospitalization/prolongation (of) | Percutaneous coronary intervention | Unlikely |
| Other medically important condition | Unconfirmed TIA | Unlikely |
| Hospitalization/prolongation (of) | Hospital admission for management of chronic condition | Unlikely |
| Hospitalization/prolongation (of) | Migraine | Not related |
| Hospitalization/prolongation (of) | Surgery unrelated to the intervention | Not related |
| Hospitalization/prolongation (of) | Anxiety/Depression | Not related |
| Hospitalization/prolongation (of) | Operation unrelated to the intervention | Not related |
| Hospitalization/prolongation (of) | Fall | Not related |
| Hospitalization/prolongation (of) | Adverse reaction to vaccination | Not related |
| Hospitalization/prolongation (of) | Surgery unrelated to the intervention | Not related |
| Hospitalization/prolongation (of) | Endoscopy/colonoscopy | Not related |
| Hospitalization/prolongation (of) | Patent foramen ovale surgery | Not related |
| Hospitalization/prolongation (of) | Patent foramen ovale surgery | Not related |

SAE = serious adverse event, TIA = transient ischaemic attack

Supplemental Table 3

Recorded Adverse Events

| **Nature of the AE** | **Relatedness of the AE to the intervention** |
| --- | --- |
| Lightheadedness and fatigue | Definitely |
| Lightheadedness and hypertension | Definitely |
| Dizziness/light-headedness | Definitely |
| Back pain | Definitely |
| Rolled ankle | Definitely |
| Exacerbation of knee arthritis | Probable |
| Episode of nausea | Probable |
| Back pain | Probable |
| Dizziness/light-headedness | Probable |
| Chest pain | Probable |
| Palpitations | Probable |
| Viral illness | Probable |
| Back pain | Probable |
| Exacerbation pelvic pain | Probable |
| Shoulder pain | Probable |
| Foot pain | Probable |
| Lower limb pain | Possible |
| Chest pain | Possible |
| Dizziness/Light-headedness | Possible |
| Palpitations | Possible |
| Dizziness/Light-headedness | Possible |
| Foot pain, swelling and bruising | Possible |
| Pain with breathing | Possible |
| Exacerbation of knee pain | Possible |
| Exacerbation of chronic vascular condition | Possible |
| Exacerbation of shoulder pain | Unlikely |
| Neck pain | Unlikely |
| Arthritis flare | Unlikely |
| Tachycardia | Unlikely |
| Breathlessness | Unlikely |
| Chest pain | Unlikely |
| Exacerbation of back pain | Unlikely |
| Chest pain | Unlikely |
| Fainted | Unlikely |
| Neck pain | Unlikely |
| Sprained ankle | Unlikely |
| Brain fog | Unlikely |
| Abdominal cramping/pain | Unlikely |
| Exacerbation of asthma | Unlikely |
| Skin abscess | Unlikely |
| Anxiety/Depression | Not related |
| Swallowing difficulty/choking | Not related |
| Viral illness | Not related |
| Procedure for nail avulsion | Not related |
| Radiotherapy treatment | Not related |
| Carpal Tunnel Syndrome | Not related |
| Fall | Not related |
| Gout | Not related |
| Tooth infection requiring extraction | Not related |
| Viral illness | Not related |
| Viral illness | Not related |
| Back injury | Not related |
| Viral illness | Not related |
| Musculoskeletal injury | Not related |
| Constipation and urinary flow issues | Not related |
| Diarrhea | Not related |
| Vaccination | Not related |
| Urinary tract infection | Not related |
| Fall | Not related |
| Bone marrow biopsy | Not related |
| Bone marrow biopsy | Not related |
| COVID-19 | Not related |
| COVID-19 | Not related |
| Influenza A | Not related |
| Unconfirmed rheumatoid arthritis | Not related |
| COVID-19 | Not related |
| Ear infection | Not related |
| Back pain and graze after being injured | Not related |
| Infection | Not related |
| Migraine | Not related |
| Gastritis | Not related |
| Anxiety/Depression | Not related |
| Chest infection | Not related |
| Viral illness | Not related |
| COVID-19 | Not related |
| Viral illness | Not related |
| Fall | Not related |
| Viral illness | Not related |
| COVID-19 | Not related |
| Urinary tract infection | Not related |
| Knee injury | Not related |
| Temporary loss of vision | Unsure |
| Hip pain | Unsure |
| Carpal Tunnel Syndrome | Unsure |
| Abdominal cramping/pain | Unsure |

AE = Adverse Event
